# Indigenous health equity in health register ascertainment and data quality: a narrative review

**DOI:** 10.1186/s12939-022-01635-2

**Published:** 2022-03-12

**Authors:** Karen Wright, Rachel M. Tapera, N. Susan Stott, Alexandra Sorhage, Anna Mackey, Sîan A. Williams

**Affiliations:** 1 Faculty of Medical and Health Sciences, Te Kupenga Hauora Māori, University of Auckland, Auckland, New Zealand; 2 Department of Surgery, University of Auckland, Auckland, New Zealand; 3 New Zealand Cerebral Palsy Register, Starship Child Health, Auckland, New Zealand; 4 Liggins Institute, University of Auckland, Auckland, New Zealand; 5 Curtin School of Allied Health, Curtin University, Perth, Australia

**Keywords:** Indigenous health, Health equity, Health register, KAUPAPA Māori, Ascertainment, Data quality

## Abstract

**Background:**

Health registers play an important role in monitoring distribution of disease and quality of care; however, benefit is limited if ascertainment (i.e., the process of finding and recruiting people on to a register) and data quality (i.e., the accuracy, completeness, reliability, relevance, and timeliness of data) are poor. Indigenous peoples experience significant health inequities globally, yet health data for, and about, Indigenous peoples is often of poor quality. This narrative review aimed to (i) identify perceived barriers for the ascertainment of Indigenous peoples on health registers, and (ii) collate strategies identified and used by health registers to support comprehensive ascertainment and high-quality data for Indigenous peoples.

**Methods:**

A Kaupapa Māori theoretical framework was utilized to guide this work. Four electronic databases were systematically searched for original articles and screened for eligibility. Studies involving health registers with Indigenous population(s) identified were included if either ascertainment or data quality strategies were described. Data extraction focused on the reporting of research involving Indigenous peoples using the CONSIDER checklist domains, ascertainment, and data quality.

**Results:**

Seventeen articles were included spanning publication between 1992 and 2020. Aspects of four of eight CONSIDER domains were identified to be included in the reporting of studies. Barriers to ascertainment were themed as relating to ‘*ethnicity data collection and quality’*, ‘*systems and structures’*, ‘*health services/health professionals’*, and ‘*perceptions of individual and community-level barriers*’. Strategies to support ascertainment were categorized as ‘*collaboration*’, ‘*finding people’*, and ‘*recruitment processes*’. Categorized strategies to support data quality were ‘*collaboration*’, ‘*ethnicity data collection and quality*’, ‘*systems-level strategies’*, and ‘*health service/health professional-level strategies*’.

**Conclusions:**

Poor-quality data for Indigenous peoples in health registers prevents the achievement of health equity and exemplifies inaction in the face of need. When viewed through a critical structural determinants lens, there are visible gaps in the breadth of strategies, particularly relating to the inclusion of Indigenous peoples in health register and research governance, and actions to identify and address institutional racism. Indigenous led research, meaningful collaboration, and a sharing of knowledge and experiences between health registers is recommended to enable research and health registers that support Indigenous self-determination and health equity.

## Background

Significant health inequities in life expectancy at birth, mortality, and morbidity exist for Indigenous peoples in Aotearoa New Zealand (NZ) and many other Indigenous populations around the world [1]. Despite these pervasive and persisting inequities, health data related to Indigenous peoples are often inconsistent, irrelevant and of poor quality [2]. Health registers, standardised datasets relevant to a health condition (e.g., cerebral palsy, cancer, rheumatic fever), collect demographic and clinical information from registered participants. As such, they differ from administrative datasets and play an important role in answering specific health questions and monitoring distribution of disease and quality of care [3, 4]. However, poor ascertainment and data quality limit potential benefit to Indigenous health and equity.

Health inequities, differences in health that are unnecessary, avoidable, unfair and unjust [5], are complex and multifactorial. Social and economic policies act as structural drivers, shaping access to the conditions of daily living [6] and, therefore, creating stratification and social class divisions. Ethnicity is one such social status category, created by socio-political environments and racism [7, 8]. Ethnicity as a biological determinant of health has been rejected [8] and ethnic inequities are theorised to be produced by three main pathways: (i) differential access to social determinants of health, (ii) differential access to health care, and (iii) differences in quality of care received [9]. Williams & Mohammed (2013) [10] describe how pathways to inequities are driven by basic determinants including biology, geographic origins, societal institutions, and importantly, by racism and discrimination.

Racism, a form of oppression based on beliefs, attitudes and behaviours concerning differences between groups defined by ethnicity [11] operates at multiple levels. Institutional racism is defined as “differential access to the goods, services, and opportunities of society by race” (Jones 2000, p1212) [7] and, being the most fundamental level, is a basic determinant of ethnic inequities. For Indigenous peoples, colonisation and colonial systems act as the underlying driver of pathways to inequities, creating power structures, policies and attitudes that advantage non-Indigenous peoples and disadvantage Indigenous peoples [12]. Health organizations have an obligation to decolonise systems and services to contribute towards achieving health equity.

The New Zealand Cerebral Palsy Register has partnered with researchers from Te Kupenga Hauora Māori, University of Auckland to support Māori health equity centred research. The aims of this study were to identify commonly perceived barriers for the ascertainment of Indigenous peoples on health registers, and to collate strategies identified and used by health registers for supporting comprehensive ascertainment and the achievement of high-quality data for Indigenous peoples on their registers. Māori are the Indigenous people of Aotearoa NZ and a Kaupapa Māori theoretical framework was utilised to guide this work. Kaupapa Māori is a culturally defined and determined approach, supporting critical, transformational, and empowering research that is ‘by’, ‘with’, and ‘for’ Māori [13–15]. The principal investigator is Māori; co-investigators are Shona and non-Māori non-Indigenous.

## Methods

An adapted version of a Kaupapa Māori narrative review framework, Ngā Poutama Whetū, provided the framework for both a systematic and critical Indigenous perspective [16]. Ngā Poutama Whetū, translated to ‘stairway to the stars’, examines power relations and privileges Māori perspectives in order to “counter the privileged mono-cultural voice within academic literature” (Hapeta, Palmer & Hermansson, 2019, p210) [16]. Methods are described below under the following headings: Kaupapa, Tino rangatiratanga, Kia piki i ngā raruraru o te kainga, Ako, Taonga tuku iho, Whānau, and Kaupapa.

### Kaupapa: collective aims and aspirations for Māori

The Kaupapa stage identifies the study parameters, which, for this study focused on two aspects of health registers relevant to Indigenous health and equity: (i) ascertainment (i.e., the process of finding and recruiting people on to a register) and (ii) data quality. For the purpose of this study, ‘data quality’ refers to accuracy, completeness, reliability, relevance, and timeliness – an adaptation of the six dimensions of data quality described by Kerr, Norris & Stockdale [17]. Indigenous people are characterised by the United Nations Declaration on the Rights of Indigenous Peoples (UNDRIP) “working definition”, recognising that there is no internationally agreed upon definition of Indigenous peoples [18].

### Tino rangatiratanga: self-determination

The autonomy of researchers is identified in this stage and demonstrated throughout the study methods. The research team determined the databases, search terms, and inclusion and exclusion criteria as relevant to the review’s Kaupapa and is outlined below.

### Kia piki ake i ngā raruraru o te kainga: socioeconomic mediation

This stage identifies whose and what knowledge counts as valid and legitimate. An electronic research database search was completed by one author on 2 February 2021 for original publications (including editorials and opinion pieces) within the following databases: Ovid MEDLINE, Scopus Elsevier, EMBASE, and PubMed. Acknowledging that specificity would be reduced, the search terms ‘Indigenous’ and ‘register’ along with related terms specific to each database (i.e., Aborigine, American Indian, First Nation, Inuit, Māori, Native American, Sami, Torres Strait Islanders) were used to conduct a wide search of potential publications but was refined to also include ‘ascertainment’ and ‘data quality’ for Ovid MEDLINE to narrow the number of articles returned from *n* = 4,479.

### Ako: culturally preferred pedagogies

Ako identifies alignment of research ‘by’, ‘with’, and ‘for’ Māori. In this review, the research team recognised that research ‘by’ and ‘with’ Māori and Indigenous peoples was likely to be limited in this research domain. As such, findings that are ‘for’ Indigenous peoples were included and the involvement of Indigenous peoples in research included as a data variable. Data were extracted using the eight domains (governance, prioritization, relationships, methodologies, participation, capacity, analysis and interpretation, and dissemination) of the CONSIDER (consolidated criteria for strengthening reporting of health research involving Indigenous peoples) checklist described by Huria et al. [19], and presented in Table 1.

**Table 1:** CONSIDER statement checklist of items to include when reporting health research involving Indigenous Peoples [19]

Item Checklist item	
Governance	
1	Describe partnership agreements between the research institution and Indigenous-governing organization for the research, (e.g., Informal agreements through to MOU (Memorandum of Understanding) or MOA (Memorandum of Agreement))
2	Describe accountability and review mechanisms within the partnership agreement that addresses harm minimization
3	Specify how the research partnership agreement includes protection of Indigenous intellectual property and knowledge arising from the research, including financial and intellectual benefits generated (e.g., development of traditional medicines for commercial purposes or supporting the Indigenous community to develop commercialization proposals generated from the research)
Prioritization	
4	Explain how the research aims emerged from priorities identified by either Indigenous stakeholders, governing bodies, funders, non-government organization(s), stakeholders, consumers, and empirical evidence
Relationships (Indigenous stakeholders/participants and research team)	
5	Specify measures that adhere and honor Indigenous ethical guidelines, processes, and approvals for all relevant Indigenous stakeholders, recognizing that multiple Indigenous partners may be involved, e.g., Indigenous ethics committee approval, regional/national ethics approval processes
6	Report how Indigenous stakeholders were involved in the research processes (i.e., research design, funding, implementation, analysis, dissemination/recruitment)
7	Describe the expertise of the research team in Indigenous health and research
Methodologies	
8	Describe the methodological approach of the research including a rationale of methods used and implication for Indigenous stakeholders, e.g., privacy and confidentiality (individual and collective)
9	Describe how the research methodology incorporated consideration of the physical, social, economic and cultural environment of the participants and prospective participants. (e.g., impacts of colonization, racism, and social justice). As well as Indigenous worldviews
Participation	
10	Specify how individual and collective consent was sought to conduct future analysis on collected samples and data (e.g., additional secondary analyses; third-parties accessing samples (genetic, tissue, blood) for further analyses)
11	Described how the resource demands (current and future) placed on Indigenous participants and communities involved in the research were identified and agreed upon including any resourcing for participation, knowledge, and expertise
12	Specify how biological tissue and other samples including data were stored, explaining the processes of removal from traditional lands, if done, and of disposal
Capacity	
13	Explain how the research supported the development and maintenance of Indigenous research capacity (e.g., specific funding of Indigenous researchers)
14	Discuss how the research team undertook professional development opportunities to develop the capacity to partner with Indigenous stakeholders?
Analysis and interpretation	
15	Specify how the research analysis and reporting supported critical inquiry and a strength-based approach that was inclusive of Indigenous values
Dissemination	
16	Describe the dissemination of the research findings to relevant Indigenous governing bodies and peoples
17	Discuss the process for knowledge translation and implementation to support Indigenous advancement (e.g., research capacity, policy, investment)

To ensure benefit, data that were deficit framing of Māori and Indigenous peoples or culture was excluded from analysis. Deficit framing focuses on Indigenous peoples as the problem [20] and identifies internal deficiencies, such as ability, motivation and behaviour, as cause of disparities [21]. Five pieces of data attributed inequitable health and health care access outcomes to the values and behaviour of Indigenous individuals and communities and were excluded from analysis.

### Taonga tuku iho: treasures to pass on

The *Taonga tuku iho* stage included the two-step appraisal and evaluation of included articles with Kaupapa-aligned inclusion and exclusion criteria. Inclusion criteria included focusing on a health register, Indigenous population(s) identified, and either ascertainment or data quality strategies described. Theses and dissertations were excluded, in addition to data that were deficit framing, as previously described.

Title and abstract screening were completed independently by two reviewers (RT and SW) with conflicts resolved by the research team. All full text articles were then read in full by two researchers (RT and either SW or KW) for suitability for inclusion in alignment with the research aims and the set inclusion/exclusion criteria.

### Whānau: extended family structure

Whānau represents the analytical stage of the review. Data were extracted under six predetermined variables: description of register, Indigenous population(s), Indigenous involvement in research, barriers to ascertainment, ascertainment strategies, and strategies supporting data quality. Consistent with thematic analysis as described by Braun & Clarke [22], data were coded (whānau—family), inter-relatedness identified (Hapū – sub-tribe), and categorised into themes (Iwi—tribe).

### Kaupapa: collective aims and aspirations for Māori

Wide dissemination supports the translation of findings into meaningful change and benefit for Indigenous peoples. The final stage of the review includes reconnecting with the Kaupapa and dissemination of findings through publication, and presentation and reporting to health register stakeholders. This study was reported in accordance with the CONSIDER statement, used to strengthen the reporting of health research involving Indigenous peoples [19].

## Results

### Study characteristics

**Fig. 1:**
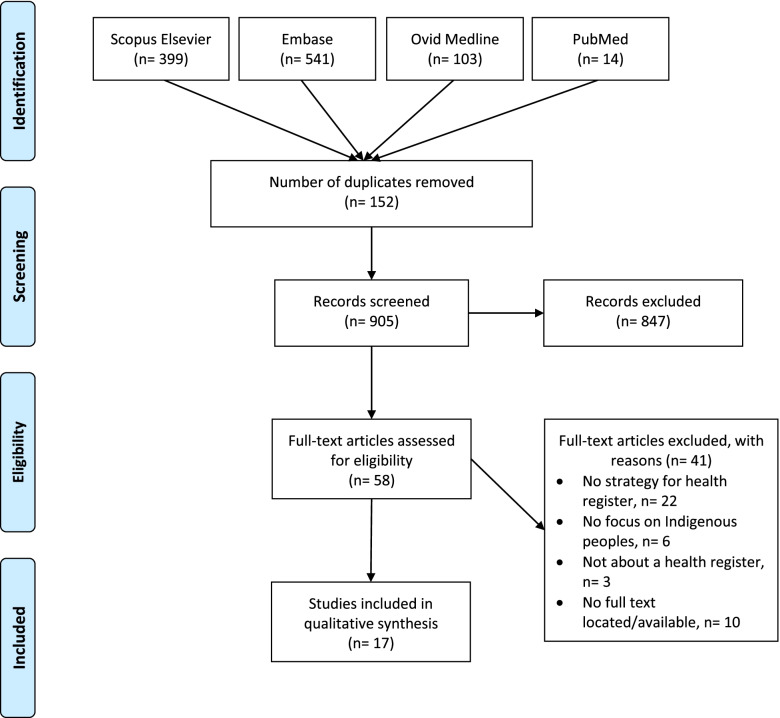
PRISMA flow diagram

A total of 1,057 records were initially identified, with 905 (all in English) being screened for eligibility after removal of duplicates, and 58 articles included for full text review (Fig. 1). Seventeen articles were finally included in this review, spanning publication between 1992 and 2020. Of the 17 studies included, the majority were based in North America (*n* = 12) followed by Aotearoa NZ (*n* = 3) and Australia (*n* = 1). One article included global Indigenous populations [23]. Full study characteristics are outline in Table 2.

**Table 2:** Characteristics of included articles, ordered chronologically. Region/country, name of registry, Indigenous populations(s), CONSIDER domain(s) identified for reporting of research involving Indigenous peoples, whether or not the study included / identified/ discussed barriers and/or strategies to ascertainment, and/or strategies supporting data quality are indicated

**Author, year**	**Region/ country**	**Name of registry**	**Indigenous Population(s)**	**CONSIDER domain(s)**	**Ascertainment**		**Data quality strategies**
					**Barriers**	**Strategies**	
Lieb et al., 1992 [24]	Los Angeles, USA	Los Angeles County AIDS Surveillance Registry	Indigenous American and Indigenous Alaskan	Prioritization, relationships	Yes		Yes
Wiggins 1996 [25]	USA	Cancer registries (non-specific)	Indigenous American	Prioritization			Yes
Dannenbaum et al., 1999 [26]	James Bay, Canada	Cree Board of Health and Social Services of James Bay Diabetes Registry	Cree of Eeyou Istchee	Prioritization, relationships		Yes	Yes
Becker et al., 2002 [27]	Portland Area, USA	Oregon State Cancer Registry, the Washington State Cancer Registry, and the Cancer Data Registry of Idaho	Indigenous American and Indigenous Alaskan	Not identified	Yes		Yes
Espey et al., 2008 [28]	USA	49 state cancer registries	Indigenous American and Indigenous Alaskan	Prioritization	Yes		Yes
Perdue et al., 2008 [29]	Indian Health Service regions (Alaska, Pacific Coast, Northern Plains, Southern Plains, Southwest, and East), USA	National Program of Cancer Registries	Indigenous American and Indigenous Alaskan	Prioritization	Yes	Yes	
Shaw et al., 2009 [30]	New Zealand	Cancer Registry	Māori	Prioritization	Yes		Yes
Johnson et al., 2009 [31]	Detroit, USA	National Program of Cancer Registries	Indigenous American and Indigenous Alaskan	Prioritization, relationships, participation	Yes		Yes
Hoopes et al., 2010 [32]	Washington State, USA	Northwest Tribal Registry, Washington State Cancer Registry	Indigenous American and Indigenous Alaskan	Governance, prioritization, relationships, participation	Yes		Yes
Zhang et al., 2011 [33]	Australia	Eight Australian cancer registries	Indigenous Australians	Prioritization	Yes		Yes
Hoopes et al., 2012 [34]	Portland IHS administrative area (Idaho, Oregon, and Washington), USA	Idaho, Oregon, Washington Cancer Registries	Indigenous American and Indigenous Alaskan	Governance, prioritization, relationships, participation		Yes	Yes
Creswell et al., 2013 [35]	Wisconsin, USA	State cancer registry	Indigenous American and Indigenous Alaskan	Governance, prioritization, relationships	Yes	Yes	Yes
Page et al., 2017 [36]	New Zealand	Australia and New Zealand Dialysis and Transplant Registry	Māori	Prioritization, relationships	Yes		Yes
Boden-Albala et al., 2017 [37]	Alaska	Alaska Native Stroke Registry	Indigenous Alaskan	Governance, prioritization, relationships		Yes	Yes
Scott et al., 2018 [38]	New Zealand	Waikato Trauma Registry	Māori	Prioritization, relationships	Yes		Yes
Layne et al., 2019 [39]	USA	State cancer registries	Indigenous American and Indigenous Alaskan	Prioritization	Yes		Yes
Diaz et al., 2020 [23]	Global	International Association for Cancer Registries	Global Indigenous populations	Prioritization	Yes	Yes	Yes

Aspects of at least one of four CONSIDER checklist domains were identified in each of the included articles. All articles identified how research aims emerged, therefore fulfilling the prioritization domain. Research aims emerged from community-based organizations[24] and empiric evidence [23, 25, 26, 28–39]. However, the inclusion of Indigenous stakeholders in the prioritization process was not identified. The relationship domain was identified in 10 articles and included authors from Indigenous health services [24, 26, 27], approval from Indigenous organizations or boards specifically identified to have Indigenous members [26, 27, 31, 32, 37], tribal access to local level data [34], Indigenous team members [35], involvement of Indigenous research units [36–38], and Indigenous advisory groups [37]. Governance was identified in three articles, specifically partnership with Indigenous health organizations [32, 34, 35]. Finally, participation was identified in three articles in terms of resource demands placed on Indigenous peoples. This included involvement of personnel in the research process [31, 32] and training [27], and grant support from Indigenous health organisations [32, 34].

Of the 17 articles, 13 included barriers to ascertainment [23, 24, 27–33, 35, 36, 38, 39]; strategies to support ascertainment and data quality were identified in six [23, 26, 29, 34, 35, 37] and 16 articles respectively [23–28, 30–39]. Both barriers and strategies to ascertainment were identified in three studies [23, 29, 35], two of which also identified data quality strategies [23, 35]. Details of the barriers and strategies are expanded below.

### Barriers to ascertainment

Four overarching themes (iwi) were identified as barriers to ascertainment of Indigenous peoples on health registers: *ethnicity data collection and quality, systems and structures, health services / health professionals,* and *perceptions of individual and community barriers* (summarised in Table 3).

1. Ethnicity data collection and quality
  Of the 13 articles [23, 24, 27–33, 35, 36, 38, 39] where barriers to the ascertainment of Indigenous peoples to health registers were identified, ethnicity data collection and quality was identified as a barrier in most (*n* = 9). Incomplete data on Indigenous status was identified as a barrier in three articles [23, 33, 38], resulting from failure to collect multiple ethnicities, variable collection of ethnicity or Indigenous status, and poor-quality ethnicity data from contributing data sources (i.e., laboratory data, administrative datasets). Inconsistent data was identified as a barrier in three articles [24, 30, 39], resulting from non-systematic ethnicity collection processes. Inaccurate data was recognised as a barrier in five articles [23, 27, 28, 32, 33], and resulted in misclassification of ethnicity through use of other data sources with poor ethnicity data quality, and inappropriate ethnicity data collection practices such as blood quantum, using name, religion, or geographical location as a proxy for ethnicity.
2. Systems and structures
  Five articles describe barriers at a ‘systems and structures’ level [23, 29, 31, 32, 36], including i) limitations in ethnicity collection systems (i.e., non-systematic and inconsistent processes, information systems unable to record all ethnicity responses, inconsistent with standard ethnicity data protocols) [23, 31, 32, 36] and data information systems [23] (i.e., ability to record and transfer information), ii) a lack of other standard protocols [29], and iii) legislation preventing collection of Indigenous status (related to historical and current socio-political recognition of Indigenous peoples) [23].
3. Health services / health professionals
  Three articles discussed barriers relating to health services and health professionals (both clinical and non-clinical staff, i.e., professional staff) [23, 29, 35], including; staff capability (i.e., ability to collect Indigenous status [23] and insufficient training [35]), staff capacity [23, 29, 35], limited availability of services [29], insufficient funding [29], and the perception that collecting Indigenous status was not important [23].
4. Perceptions of individual and community-level barriers
  From the perspective of authors of included articles, individual and community level barriers were identified as potential barriers in three articles [23, 24, 29] including discrimination by ethnicity [23, 24] and the accessibility of services [29].

**Table 3:** Summary of key Themes (Iwi, in Italics) and the inter-related codes (Hapū, in dot points)

**Barriers to ascertainment of Indigenous peoples**	**Strategies to support ascertainment of Indigenous peoples**	**Strategies to support data quality for Indigenous peoples**
*Ethnicity data collection and quality* • Incomplete • Inconsistent • Inaccurate	*Collaboration* • Engaging with Indigenous peoples • Engaging with existing health systems	*Collaboration* • Engaging and involving Indigenous peoples and organisations • Engaging with other organisations • Data linkage
*Systems and structures* • Ethnicity data collection and data information systems • Legislation • Lack of standard protocols	*Finding people* • Raise community awareness • Recruit from Indigenous health providers • Legislation	*Ethnicity data collection and quality* • Standard ethnicity protocols • Self-reported ethnicity • Validation
*Health services / health professionals* • Staff capability and capacity • Availability of services • Adequate funding • Collecting Indigenous status not prioritised	*Recruitment processes* • Staff training • Indigenous language speaking staff • Available enrolment resources	*Systems-level strategies* • Information systems • Standard protocols and processes • Reporting and publications • Registry standards • Quality assurance plan
*Perceptions of individual and community-level barriers* • Discrimination • Accessibility of services		*Health service / health professional-level strategies* • Adequate resource • Responsive protocols • Staff capability • Staff feedback

### Ascertainment strategies

Three overarching themes (iwi) were identified from strategies supporting ascertainment of Indigenous peoples on health registers: *collaboration, finding people, and recruitment processes* (Summarised in Table 2).

1. Collaboration
  Four articles included strategies involving working with other groups including Indigenous leaders [26], Indigenous communities [29, 34] (i.e., education forums, support groups) and existing health systems [37] (i.e,. integrating registries into existing health systems).
2. Finding people
  Four articles described strategies related to ‘finding people’ to enrol in a health register including raising community awareness through media and Indigenous leaders [26], recruiting from Indigenous health providers [34, 35], and legislation mandating collection of Indigenous status [23].
3. Recruitment processes
  Four﻿ articles described strategies around the registration process itself including staff training [23, 35], Indigenous language speaking staff [26], and making enrolment resources available [34].

### Strategies supporting data quality

Nearly all articles identified strategies to support data quality (*n* = 16) [23–28, 30–39], with the following four themes (Iwi) identified: *collaboration, ethnicity data collection and quality, systems-level strategies,* and *health service / health professional-level strategies.*

1. Collaboration
  Strategies categorised as ‘collaboration’ included engaging with Indigenous health providers to foster reciprocal reporting relationships [35], and with Indigenous peoples in the development [25] and governance of registries [23], supporting appropriate and ethical collection and use of data. Collaboration with both tribal and urban Indigenous health services supported improved data quality [32], including through direct reporting from clinics to health registers [35]. Non-Indigenous health organisations [31, 32], other registers [25, 28, 34], and international strategic networks [23] were also recognised to support data quality. Data linkage was identified as a strategy to improving ethnicity data [31, 32], specifically using census data [30, 33], hospital data [33], Indigenous health service data [28, 39], and tribal enrolment data [23, 34]. Furthermore, collaboration between tribes, states, and academic institutions regarding data linkage was recognised as supporting both data quality and trusting relationships [31].
2. Ethnicity data collection and quality
  Strategies were identified as supporting quality ethnicity data in seven articles [24, 28, 30, 32, 33, 36, 39] including standardised processes (i.e., using standard ethnicity protocols [24, 30, 33, 36] and uniform data collection tools [28], collecting Indigenous status on laboratory forms [33], recording ethnicity data source [33], auditing Indigenous status [33]), collecting self-reported ethnicity [32, 36, 39], and validating ethnicity [24, 39].
3. Systems-level strategies
  System-level strategies were described in just less than half of included articles (*n* = 8) [23, 25, 26, 30, 32, 37–39]. Information systems supported quality data and were recognised as requiring appropriate safeguards[26], being easy to use [25, 26], and integrated to enable data linkage [23]. Standard protocols [26, 38, 39] and processes [38], such as multiple data collection points [37] and effective point of care data collection [23], were identified to support quality data collection. System-level strategies were inclusive of reporting, specifically regular reporting [26, 30, 38], regular evaluation [26], the reporting of Indigenous data [23], and reporting through publications and annual auditing [26]. Related to standard protocols was registry standards [32] and a formal quality assurance plan [30].
4. Health service / health professional-level strategies
  Health service-level strategies were predominantly related to adequate funding and financial commitment [23, 25, 26] but also included responsive and tailored protocols to address regional variation in barriers [23]. Health professional-level strategies focused on staff capability, particularly staff training [38] around ethnicity [27, 30], and capturing relevant information [23]. Skilled staff supported high quality data [25]. Staff feedback was described as an approach to identify and incorporate improvement recommendations [26], with representation across the data system [23].

## Discussion

This study provides a comprehensive overview of commonly perceived barriers and proposed strategies supporting ascertainment and high-quality data of Indigenous peoples on health registers. Multiple interventions at health professional, service and system levels have been identified and may provide guidance for health registers seeking to prioritize health equity for Indigenous peoples.

Of note, poor ethnicity data quality was identified as a significant barrier to ascertainment and high-quality ethnicity data central to the achievement of high-quality data for Indigenous peoples. The existence of poor-quality ethnicity data in health registers is, unfortunately, unsurprising, with Indigenous population data stated to often be inconsistent, irrelevant, of poor quality, produced in an environment of mistrust, and controlled by those external to Indigenous nations [2]. Subsequently, data often undercounts Indigenous peoples, does not accurately reflect Indigenous realities, and does not inform Indigenous peoples’ needs [2]. For health registers, these critical data issues limit the potential to identify, prioritize, and address Indigenous health and health service-related inequities.

Despite the breadth of barriers identified for ascertainment, there are noticeable gaps in the barriers and pathways to inequities when a socioecological framework, such as that proposed by Williams [8] is applied. Barriers identified include some basic causes (e.g., legislation) and proximal pathways (e.g., discrimination, access to health services, and health service orientation). However, racism, specifically institutional racism, and broader political and economic institutions are noticeably absent.

Findings from this review indicate a disconnect between perceived barriers and solutions. Strategies to support ascertainment and data quality are predominantly orientated towards individual responses and proximal pathways, thus focussing on access to services, general processes/systems, and individual or community responsibility. Although important, such strategies are unlikely to eliminate ethnic inequities if fundamental causes, such as institutional racism, are left unaddressed [10, 40].

The reporting of the CONSIDER checklist [19] for health research involving Indigenous peoples provides potential insight into the degree of collaboration between health registers and Indigenous peoples and organizations. Few studies included within this review overtly described governance and partnership agreements with Indigenous organizations, suggesting either absence in existence or in reporting. An absence of Indigenous leadership or Indigenous participation in Indigenous health research is common, raising significant concerns regarding the appropriateness of approach, methods, interpretation and reporting, and prioritizing of health research itself [41]. Indigenous methodologies, strength-based analysis and interpretation, activities to support Indigenous research capacity, and dissemination to Indigenous governing bodies were also not explicitly identified within the studies included within this review. Favourable research impact and benefit is more likely when issues are relevant to Indigenous peoples, Indigenous peoples are participants, Indigenous knowledges and perspectives are incorporated, findings meaningful, and potential end-users engaged from the outset [42]. Importantly, benefit from Indigenous research must be meaningful and valued by diverse Indigenous communities [43].

In contrast, collaboration with Indigenous communities and organizations in the development and governance of health registries was identified as a potential strategy supporting Indigenous health equity. This finding is consistent with Indigenous data sovereignty scholarship articulating both the right and need for Indigenous knowledges and approaches to be integrated into policy and practice [44]. Indigenous data sovereignty is defined as the right of a nation to “… control the collection, ownership, and application of its own data” (US Indigenous Data Sovereignty Network) [45]. Importantly, it is derived from inherent rights of Indigenous peoples to govern their peoples, lands, and resources, and is inclusive of data from and about Indigenous peoples, resources and environments [44]. Furthermore, it provides the opportunity to enhance community trust in data and improve data availability, quality, and relevance to support population health gain [2, 46].

Through colonial practices, including the suppression of Indigenous knowledge systems and exclusion of Indigenous peoples from data sovereignty, Indigenous peoples have been divorced from data infrastructure and capacity into a state of “data dependency” [46]. Health registers that predominantly reflect non-Indigenous ways of knowing and doing may, although potentially unintentional, fail to identify and adequately address issues relevant to Indigenous peoples. Therefore, to support high quality Indigenous data and realise Indigenous data sovereignty in health registers, systematic and structural change is required. Three main themes supporting successful Indigenous data sovereignty have previously been identified: (i) strategic responses to data challenges; (ii) engaging with community to educate leaders and citizens about data; and (iii) using data to inform policy decisions and resource allocation to strengthen Indigenous self-determination [2]. Such transformation requires transfer of power and control [47], and both organizational and resource commitment to Indigenous health gain and equity.

This study has several strengths. A Kaupapa Māori approach and use of the Ngā Poutama Whetū framework supported a robust narrative review process that centred Māori worldviews and Indigenous health gain. The systematic approach and deliberate exclusion of deficit framing support findings that are of benefit to Indigenous health and equity. Inevitably, there are also some limitations to identify and discuss. Outcomes of this narrative review are limited to research published in English and those available and identified within the select databases. Of note, most research is in North America and, therefore, the generalisability of findings outside of North America should be considered. Few articles overtly aimed to identify barriers and strategies relating to ascertainment and data quality of Indigenous peoples on health registers. As such, an inclusive approach was used within our screening process (i.e., research articles continued through to a full text review even though no clear mention of barriers or strategies were made). Even with this inclusive and systematic approach, it is possible that further eligible studies are available that were not included within this review. In addition, as Indigenous involvement in research was limited, barriers (real or perceived) and strategies identified in this review may not reflect Indigenous peoples’ perspectives and preferences. Finally, assessing the ‘success’ or effectiveness of strategies was outside the scope of this study. There is significant opportunity for future research, led by or conducted in partnership with Indigenous researchers and organizations, to review, audit and evaluate targeted approaches to the ascertainment of Indigenous peoples and achievement of high data quality in health registers.

## Conclusion

Health registers are central to the accurate monitoring of disease prevalence and outcomes. Although there is a considerable body of peer reviewed published evidence pertaining to ascertainment and data quality of health registers, there is more limited evidence to identify strategies specific to Indigenous peoples. From the literature included in this systematic narrative review, it is apparent that multiple interventions at many levels (system, service, and community) are used to ascertain Indigenous peoples on health registers and ensure high quality data. However, when viewed through a critical structural determinants lens, there are visible gaps in the breadth of strategies, particularly the inclusion of Indigenous peoples in health register and research governance, and actions to identify and address institutional racism. These gaps perpetuate the collection of inconsistent, incomplete, and poor-quality data for Indigenous peoples in health registers, preventing the achievement of health equity and exemplifying inaction in the face of need. Recognising that, though unique barriers and strategies may exist for different Indigenous populations across the world, we propose that potential commonalities present an opportunity for Indigenous led research and a sharing of knowledge and experiences between health registers. Sharing, networking, and meaningful collaboration with Indigenous communities and organizations supports health registers to be structured and operate to achieve Indigenous health equity.

## Data Availability

The datasets analysed during the current study are available from the corresponding author on reasonable request.

## Abbreviations

IP: Indigenous people
NZ: Aotearoa New Zealand
USA: United States of America
